# Performance of a feature-based algorithm for 3D-3D registration of CT angiography to cone-beam CT for endovascular repair of complex abdominal aortic aneurysms

**DOI:** 10.1186/s12880-018-0285-1

**Published:** 2018-11-08

**Authors:** Giasemi Koutouzi, Behrooz Nasihatkton, Monika Danielak-Nowak, Henrik Leonhardt, Mårten Falkenberg, Fredrik Kahl

**Affiliations:** 1 Department of Radiology, Institute of Clinical Sciences, Sahlgrenska Academy, Gothenburg, Sweden; 2 K. N. Toosi University of Technology, Tehran, Iran; 3 Department of Electrical Engineering, Chalmers University of Technology, Gothenburg, Sweden; 4 Center for Mathematical Sciences, Lund University, Lund, Sweden

**Keywords:** Cone-beam CT, Aortic aneurysm, Image registration, Feature-based registration, Intensity-based registration

## Abstract

**Background:**

A crucial step in image fusion for intraoperative guidance during endovascular procedures is the registration of preoperative computed tomography angiography (CTA) with intraoperative Cone Beam CT (CBCT). Automatic tools for image registration facilitate the 3D image guidance workflow. However their performance is not always satisfactory. The aim of this study is to assess the accuracy of a new fully automatic, feature-based algorithm for 3D3D registration of CTA to CBCT.

**Methods:**

The feature-based algorithm was tested on clinical image datasets from 14 patients undergoing complex endovascular aortic repair. Deviations in Euclidian distances between vascular as well as bony landmarks were measured and compared to an intensity-based, normalized mutual information algorithm.

**Results:**

The results for the feature-based algorithm showed that the median 3D registration error between the anatomical landmarks of CBCT and CT images was less than 3 mm. The feature-based algorithm showed significantly better accuracy compared to the intensity-based algorithm (*p* < 0.001).

**Conclusion:**

A feature-based algorithm for 3D image registration is presented.

## Background

Since endovascular aortic repair (EVAR) for abdominal aortic aneurysm (AAA) was first described by Volodos and Parodi [1, 2] there has been a shift away from traditional open surgery towards the less invasive option of endovascular treatment. This has been facilitated by a fast evolution in stent graft design and imaging technology.

Development of fenestrated and branched stent grafts allows treatment of complex juxta-renal and supra-renal AAA [3, 4]. These devices have openings or side-branches, preserving perfusion of vital organs while excluding the aneurysm. Accurate placement of such stent grafts is crucial, not only in the proximal/distal dimension but also in the rotational dimension, matching the fenestrations and branches with the origins of the target vessels. Even after optimal placement of the main aortic device, subsequent catheterization of target vessels to deliver mating stents can be difficult. Treatment of complex AAA with fenestrated or branched EVAR is therefore challenging, and image information on the patient’s anatomy is particularly important during these procedures [5–10].

The introduction of cone-beam computer tomography (CBCT) in the interventional suite allows intraoperative acquisition of 3D images. CBCT can be done with or without contrast enhancement. When used for image fusion, CBCT is usually done without contrast, to spare the patient’s renal function. Multimodality 3D fusion and projection of selected details on the live fluoroscopy screen enables visualization of the patient’s anatomy, captured in the preoperative images, to facilitate intraoperative navigation [11, 12].

A crucial step in image fusion for intraoperative guidance is the registration of preoperative computer tomography angiography (CTA) with intraoperative CBCT [13, 14]. Registration can be done by manual alignment in multi-planar reconstruction (MPR) projections or using automatic algorithms. Manual registration is time consuming and requires a high degree of anatomical and procedural insight. In fact, the need for manual registration during the procedure may explain the still limited dissemination of image fusion to vascular centers. Automatic registration algorithms in commercially available systems are often intensity-based. However, fully automatic registration presents several difficulties. First, the fields of view (FOVs) of a CTA and a CBCT differ markedly, the CTA often being approximately three times the size of the CBCT. Secondly, the exact posture of the patient often varies between the preoperative and intraoperative image acquisitions. For example, during CT the patient lies with the knees slightly bent, whereas during the procedure the patient usually lies on the operating table with the legs straight. Thirdly, the contrast-enhanced aorta in the CTA has no image counterpart in the non-enhanced CBCT. Fourthly, the preoperative images may not be perfect with sub-millimeter slices; sometimes only thicker reconstructed slices are available. For all the reasons above, standard intensity-based algorithms for automatic 3D-3D image registration are less than perfect in the clinical situation [14].

Most of the existing CT-CBCT registration algorithms are intensity-based. Many of these techniques are different variants of the well-known Demons algorithm [15] in which a deformable grid models the non-rigid image transformation. For example, Nithiananthan et al. [16] propose a variant of the Demons algorithm in which an intensity correction step is performed on the CBCT image at every iteration of the registration algorithm. A more flexible intensity correction scheme has been proposed in by Lou et al. in 2013 [17].

The registration proposed by Yu et al. [18], is done on 3D gradient fields to deal with the intensity inaccuracies of CBCT in deformable intensity-based registration.

Perhaps the most relevant work to our application of interest is presented by Miao et al. [19], where a multi-stage CBCT to CT registration technique has been proposed for aortic stenting. First, a 2D global search technique is applied to the maximum intensity projection images to estimate an initial translation parameter. Then, the spine in two images are segmented out and rigidly registered. Finally, a deformable registration is applied to fine-tune the alignment around the aorta.

Feature-based methods have also been employed for CT-CBCT image registration. Xie et al. [20] use 3D SIFT features to map the rectal contours from the CT to the CBCT image. The mapping is done by finding a set of SIFT matches between the two volumes and computing a thin-plate spline transformation between them. Xie et al. [21] in 2011 used the same method in a two stage manner for the registration of the liver. In the first stage, the relative position of the liver volumes is found in two images. In the second stage, using manually segmented liver in the first image, a more accurate registration is performed y only exploiting the feature points inside the liver volume in both images. Paganenelli et al. [22] investigated the performance of 3D SIFT features in adaptive radiation therapy. However, the SIFT features are used to evaluate the performance of other non-rigid registration techniques, and not as a means of registration.

Here, we have developed a novel feature-based algorithm for affine 3D-3D image registration. It was first presented at the International Symposium on Biomedical Imaging, 2015 [23], where it was shown to perform well for inter-subject registration, and where both the source and target images were of the same modality (CT or MRI). In the current work, we have developed the method further by allowing more general transformations parametrized by splines in order to improve the registration accuracy in regions of soft tissue. The aim of this study was to evaluate the performance of this feature-based algorithm for 3D-3D registration of CTA to CBCT, and to compare its accuracy with that of a commercially available intensity-based algorithm that optimizes normalized mutual information.

## Methods

The algorithm proposed was evaluated offline using data from 14 clinical cases. The study has been approved by the regional research ethics committee. No formal consent was required.

One patient had a common iliac artery aneurysm and was treated with a branched iliac stent graft, and all other patients had a juxta-renal or thoraco-abdominal aneurysm and were treated with fenestrated, branched, or chimney EVAR at the hybrid operating room of Sahlgrenska University Hospital between June 2012 and March 2015 (12 men and two women with a mean age of 73.6 years (standard deviation (SD) ± 5.4)). Characteristics of the patients and procedures are given in Table 1.

**Table 1:** Characteristics of patients and procedures

Patient	Age (years)	Gender	BMI (kg/m^2^)	Aneurysm type	Aneurysm size (mm)^1^	Procedure
1	69	M	30	Common iliac artery aneurysm	40	Iliac Branched
2	82	F	34.4	Juxta-renal	62	FEVAR
3	81	M	23.2	Thoraco-abdominal	90	BEVAR
4	71	M	23.8	Juxta-renal	72	FEVAR
5	72	M	27.5	Juxta-renal	58	FEVAR
6	75	M	23.8	Juxta-renal	65	Chimney EVAR
7	76	M	24.3	Juxta-renal	65	FEVAR
8	67	M	24.7	Juxta-renal	70	FEVAR
9	67	M	25.8	Supra-renal	83	BEVAR
10	83	M	33.3	Juxta-renal	62	FEVAR
11	76	M	23.3	Juxta-renal	60	EVAR
12	69	M	24.5	Thoraco-abdominal	90	BEVAR
13	70	F	27.3	Thoraco-abdominal	100	BEVAR
14	73	M	19.6	Thoraco-abdominal	62	BEVAR

*F,* female*; M,* male*; FEVAR,* fenestrated endovascular aneurysm repair*; BEVAR,* fenestrated endovascular aneurysm repair

^1^ *Aneurysm size was defined as the maximal aortic diameter perpendicular to the line of flow*

All the patients had a pre-procedural multi-detector CTA and an intraoperative CBCT.

### CT

Throughout the study, a variety of 64-slice multi-detector spiral CT scanners from different manufacturers were used at our hospital and the referral hospitals of the region. The preoperative CTA was performed within 6 months (median 3 months, range 2 days to 6 months) before the EVAR procedure.

The CT scans were performed according to routine protocols designed for aortic imaging. The tube voltage varied between 80 and 120 kV. The contrast medium used was of non-ionic low-osmolar type with a concentration of 350 mg I/mL and an injection rate of 4–5 mL/s. The datasets available at the time of the procedures had a median slice thickness of 1.25 mm (0.7–3.0 mm).

### CBCT

All procedures were performed in the same hybrid room, which was equipped with a multi-axis robotic C-arm system and a dedicated post-processing workstation (Artis Zeego and Syngo; Siemens Healthcare GmbH, Forchheim, Germany).

A low-dose CBCT without contrast was performed at the start of the each procedure, just before vascular access. During image acquisition, the C-arm (equipped with a 30 × 40-cm flat-panel detector in either landscape or portrait orientation) rotates around the patient in a 200° trajectory. The CBCT protocol used at our institution (5 s DCT Body Care) acquires 248 projection images (0.8°/image) at a configured detector dose of 0.36 μGy. The projection images are transferred automatically to the workstation, where they are reconstructed to.

CT-like images with an isotropic voxel size of 0.5 mm. The cylindrical volume captured by a CBCT has a diameter of 25 cm and a height of 19 cm with the detector in landscape mode.

(19 cm and 25 cm, respectively, in portrait mode).

To provide the best conditions for the 3D fusion process that followed, the patient was centered on the table so that the spine was in the center and the iliac spines were visible at the.

caudal end of a frontal view, and the lumbar vertebrae were visible in the lower aspect of a lateral view.

#### Feature-based registration

Our approach for registration is based on detecting and matching features. Three-dimensional scale-invariant feature transform (SIFT) features are detected in both CT and CBCT images, and 3D SIFT descriptors are computed for each feature [24]. Each feature point in the CT images is matched to its closest feature point in the CBCT image in terms of the Euclidean distance between the feature descriptors. Similarly, each CBCT feature is matched to a CT feature. Only the feature pairs that are matched in both directions are kept and rest of the feature points are discarded. Then we run a geometry-aware RANSAC test, assuming that the correct feature locations are related by an affine transformation [25] to remove the matches that are not consistent geometrically. Due to nonlinear deformation the body undergoes, the transformation between the two image volumes is not exactly affine. The this reason we use a high threshold of 10 mm to determine the inliers in the RANSAC algorithm.

**Fig. 1:**
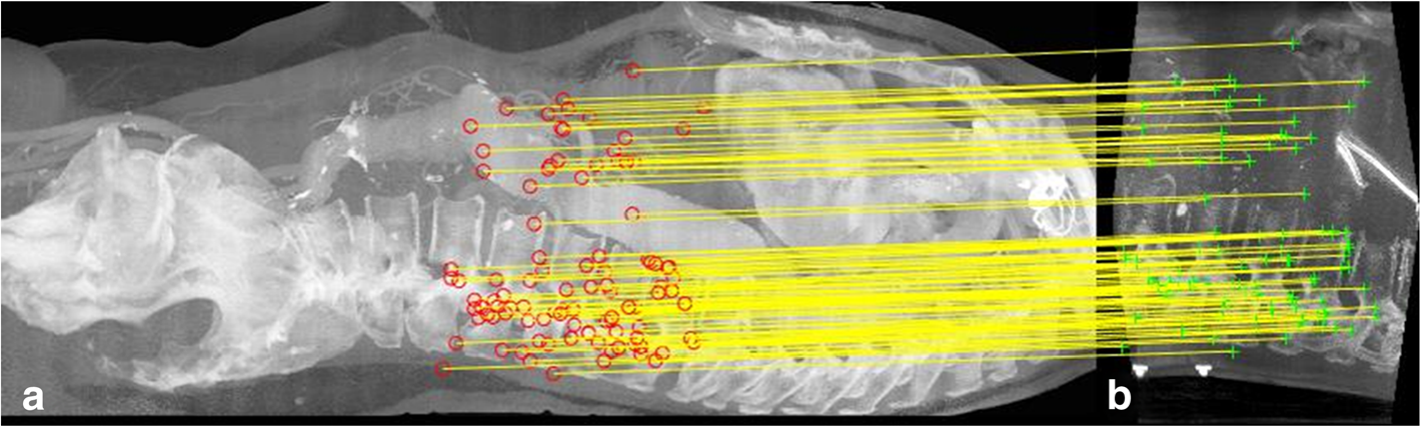
**a** CT-Angiography, **b** Cone-Beam CT: feature matching with local correspondence search. Features are obtained from the soft tissue area and from bony structures. For the sake of illustration, only 100 (randomly selected) matches are shown. Lateral views of the 3D volumes are shown using max projection

In all cases we examined on, this gave a very robust affine transformation between the CBCT and CT images. However, one major problem is that most of the matched features are located around the vertebral column. The reason is that there are a large number of features (in the order of few thousands) in both images. In the boundaries of vertebrae, there are clear structures that can be distinguished—even among thousands of features. However, in the soft tissue area, a correct match cannot easily be found among this large number of features. Still, this initial affine estimate of the registration, which aligns vertebrae, gives an accuracy of less than a centimeter even in the soft tissue area. Thus, to find more matches in the soft tissue area, given the initial affine estimate, the algorithm first transforms the CBCT into the CT image space and removes all the CT feature points located outside the boundaries of the transformed CBCT volume (plus a small margin). Then it repeats the feature-matching procedure, but this time, to find the nearest feature descriptors, it performs a local search in which each feature is only searched against the features in a 10 mm radius of its transformed location in the other image. Then the RANSAC algorithm is repeated, but this time with a 5 mm threshold. As a result, we can obtain more matches in the soft tissue area. The feature matches obtained for one CBCT-CT pair are illustrated in Fig. 1. The procedure yields a better affine transformation, as it also tries to align the soft tissue area.

**Fig. 2:**
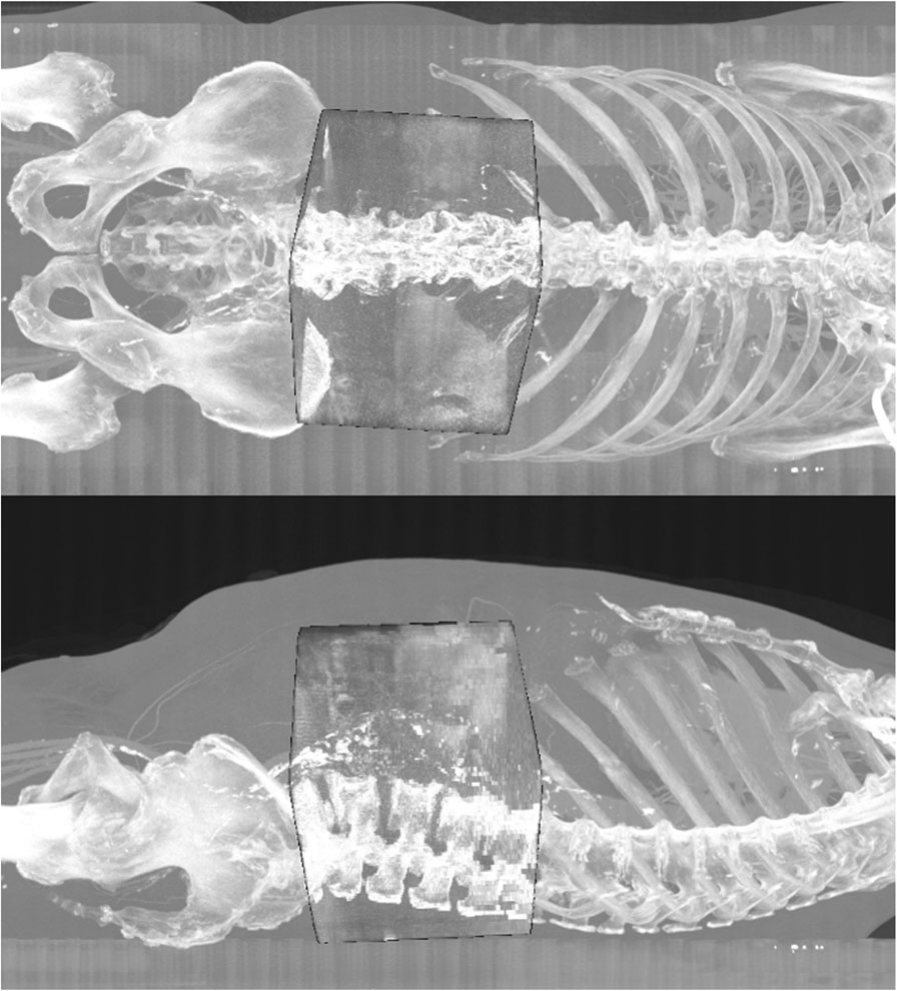
Fused CBCT-CT pair after fully automatic feature-based image registration

As a final step, the algorithm computes a thin-plate spline transformation between the two images using the feature matches. The thin-plate spline transformation is suitable for when we have an affine transformation plus a rather small nonlinear deformation. Using N three-dimensional point correspondences it gives 3 N + 12 parameters out of which 12 parameters account for a global affine transformation and 3 N parameters model the nonlinearity. This improves the registration accuracy compared to when using a global affine transformation. Fig. 2 illustrates a fused CBCT-CT pair after fully automatic feature based registration.

### Registration using the intensity-based algorithm

In order to evaluate the accuracy of the new algorithm, a comparison was made with a commercially available intensity-based, normalized mutual information algorithm (Syngo 3D3D image fusion, Siemens Healthcare), which is a standard algorithm used for 3D3D fusion of CT with CBCT.

### Measurements

Registration error was defined as the 3D distance between landmarks on CBCT and corresponding landmarks on CTA.

Six anatomical landmarks on each dataset were used for registration validation. The landmarks were vascular calcifications and bony structures that could be clearly identified in both modalities. Since the benefit of image fusion in complex EVAR procedures lies in position information concerning the ostia of the renal and the visceral arteries, the landmarks chosen were: one aortic calcification at the level of the left renal ostium, one aortic calcification at the level of the right renal ostium, and one aortic calcification at the level of the SMA ostium. The three remaining landmarks were distinct points on each of the nearby vertebrae Th12, L1, and L2.

The landmarks were first identified in the preoperative CT and then in the intraoperative CBCT. After automatic image registration, the 3D alignment error between the corresponding landmarks from the two modalities was calculated.

Measurements were performed using MATLAB for evaluation of the feature-based algorithm and a post-processing workstation (Syngo Workplace; Siemens Healthcare) connected to our angio suite for the intensity-based algorithm. For this purpose, the same landmarks had to be identified twice for each dataset.

Furthermore, for estimation of inter-observer agreement the landmarks were identified in each modality by two independent radiologists who were blind regarding each other’s landmarks and regarding the result of the registrations.

### Statistics

Continuous data are presented as median with range and as mean with SD. The Wilcoxon signed-rank test was used to test whether there was a statistically significant difference in accuracy between the feature-based algorithm and the intensity-based algorithm. The Wilcoxon signed-rank test was also used to test whether there was a significant difference in accuracy between aortic and bony landmarks for each algorithm. Inter-class correlation coefficient (ICC) (2,1) was used to estimate inter-rater reliability. Statistical analyses were conducted with SPSS for Windows version 24.0 (IBM Corp., Armonk, NY, USA). Any *p*-value < 0.05 was considered statistically significant.

Spearman’s rank correlation coefficient was used to investigate whether the slice thickness of the preoperative CT influenced the accuracy of the algorithms.

## Results

The feature-based algorithm was more robust and accurate than the intensity-based algorithm (*p* < 0.001). The median 3D target error for the feature-based algorithm was 2.3 mm (range 0.4–7.9 mm) and the median error for the intensity-based algorithm was 31.6 mm (range 0.5–112.2 mm). A 3D error of < 3 mm was found for 73% of the landmarks using the feature-based registration and for 20% of the landmarks using the intensity-based algorithm. A 3D error of < 5 mm was observed for 94% of the landmarks using the feature-based algorithm and for 28% of the landmarks using the intensity-based algorithm. The feature-based algorithm had a 3D error of < 10 mm in all cases whereas the 3D error for the intensity-based algorithm was < 10 mm for 29% of the landmarks.

**Fig. 3:**
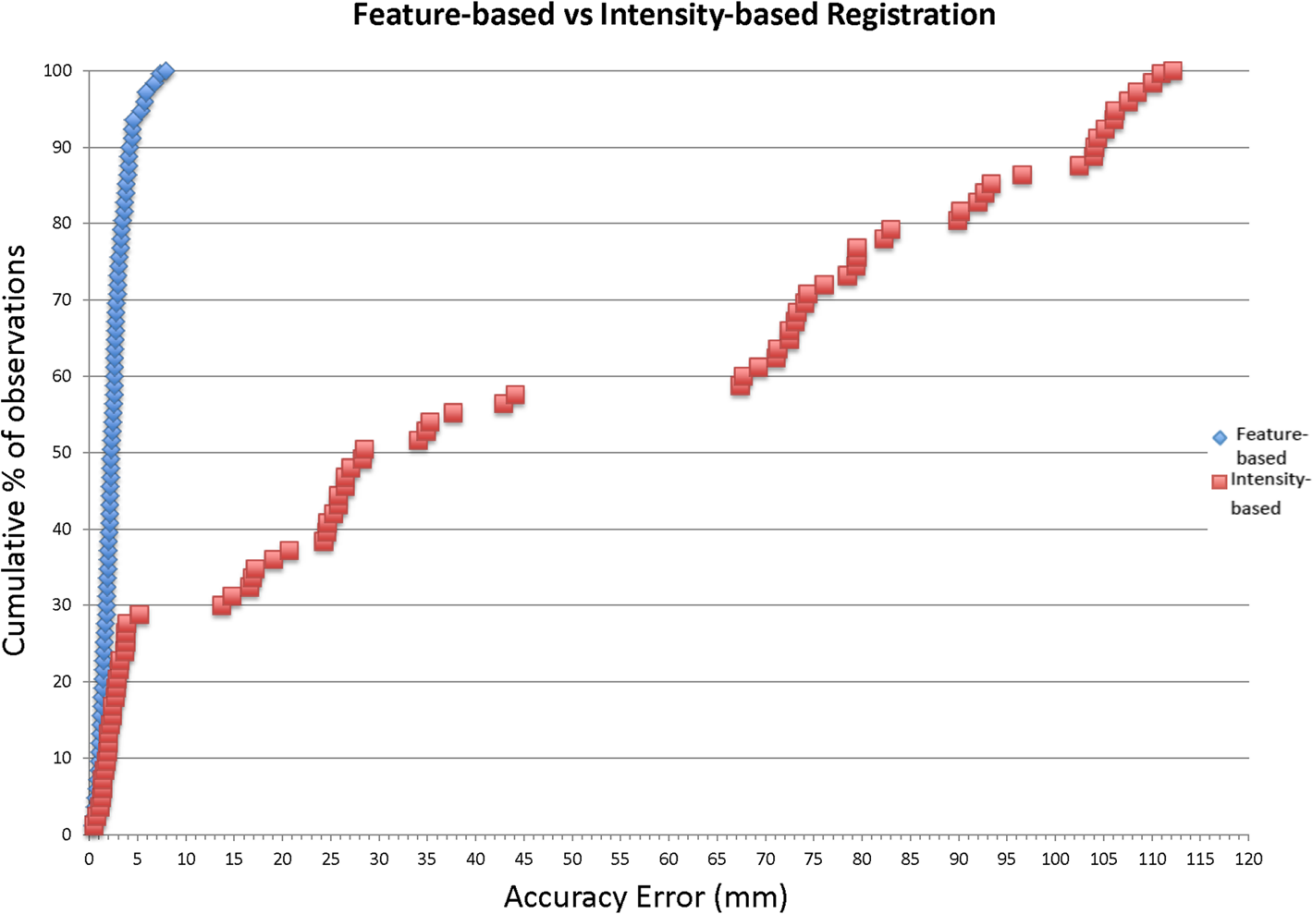
Cumulative percentage graph showing the frequency distribution of the accuracy error of each landmark for the feature-based and for the intensity-based algorithm

**Fig. 4:**
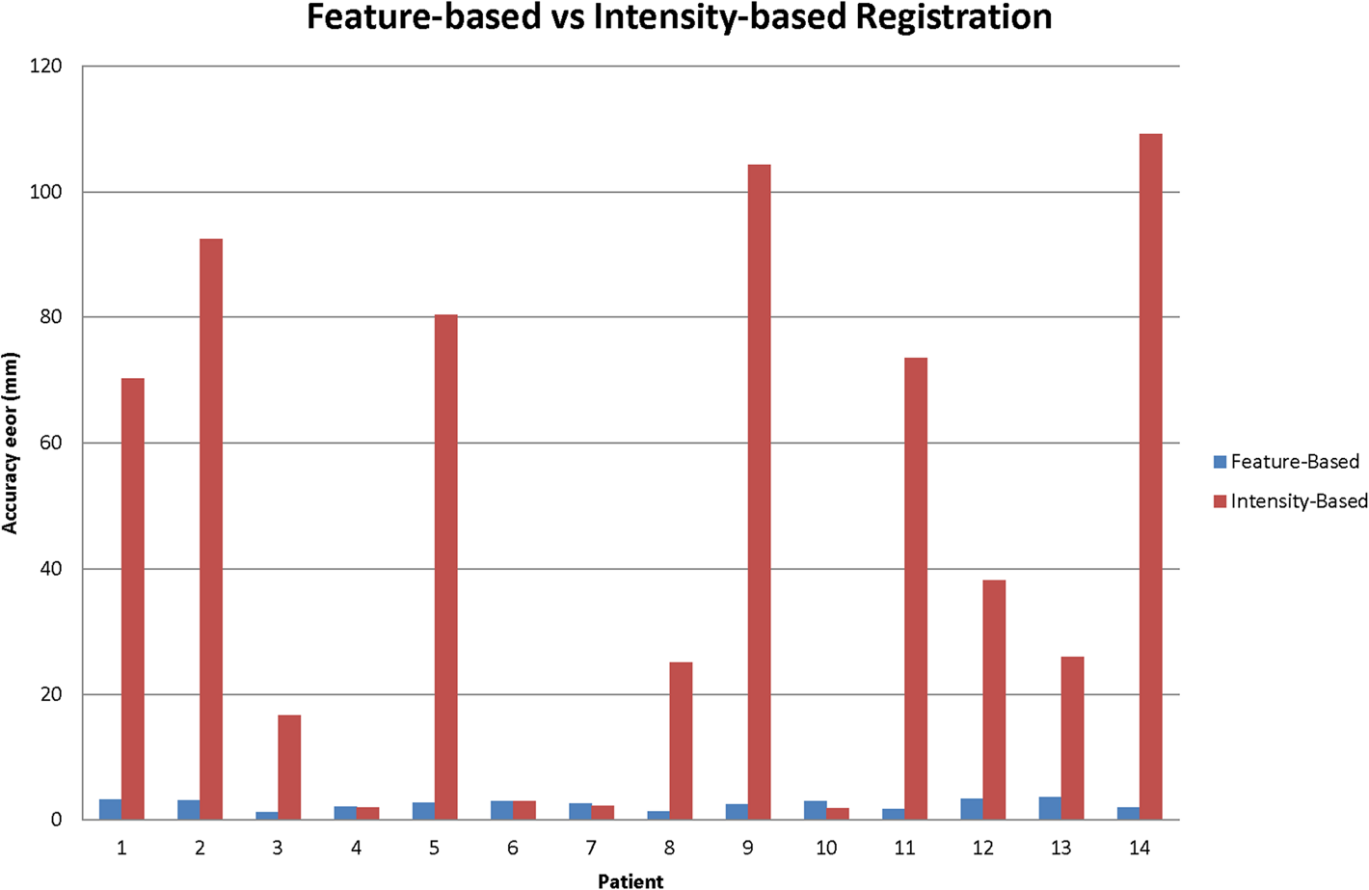
Diagram showing the average accuracy error of the feature-based algorithm and the intensity-based registration algorithm for each patient

The distribution of the 3D errors for the 84 points evaluated for each algorithm was plotted on a cumulative percentage graph (Fig. 3). Figure 4 illustrates the average 3D error for each patient.

There was no significant difference in alignment accuracy between bony landmarks and aortic calcifications in any of the two algorithms.

Correlation analysis revealed that the slice thickness of the preoperative CT had no significant influence on image fusion accuracy (intensity-based algorithm, *P* = 0.52; feature-based algorithm, *p* = 0.77).

### Reproducibility

The inter-observer agreement was almost perfect, with an ICC of > 0.8 for both algorithms.

## Discussion

In this article we present a robust, fully automatic feature-based algorithm for 3D-3D registration of CTA to CBCT. To our knowledge, this is the first study to validate a feature-based algorithm for image fusion of these modalities using clinical cases.

Image fusion has an expanding role for intraoperative guidance during endovascular repair of complex aortic aneurysms. Fenestrated EVAR (FEVAR) and branched EVAR (BEVAR) are complex and technically challenging operations, demanding precise stent graft positioning, and precise visceral and renal artery cannulation and stenting. These procedures are time consuming and involve risks of embolization and thrombosis.

In the last decade, the introduction of CBCT in radiology suites has revolutionized intraoperative image guidance. Image fusion can facilitate intraoperative guidance by overlaying important anatomical information from pre-procedural CT on the live fluoroscopic image—thus reducing procedure time, radiation dose, and the amount of contrast medium used [5–8].

However, a key determinant of widespread clinical application of the fusion technique is the ease of use and the accuracy of image registration. Manual 3D3D registration of preoperative CTA with intraoperative un-enhanced CBCT is a challenging procedure, requiring the operator to be skilled in using advanced fusion software.

Commercially available systems for automatic registration may be helpful, if they are accurate. However, these systems are still not sufficiently robust and often result in large misalignments, thus requiring inconvenient manual interaction during the procedure.

In 2016, a study on 19 EVAR cases assessed the accuracy of fully automatic registration between CT and CBCT using a feature-based mutual information algorithm. The fully automatic registration alone was not sufficient for EVAR guidance (defined as < 3 mm deviation at the lower renal artery ostium), and in 42% of the cases the deviation in registration at the lower renal artery was greater than 20 mm [14].

Schulz et al. (2016) [26] reported their experience with image fusion in a larger cohort of 101 consecutive EVARs using a two-step algorithm designed to automatically align the 3D datasets [19]. First, bony structures were aligned using normalized mutual information and then alignment of vessels and vessel calcifications of the aorta was performed in a second step. This software was included in a prototype workplace with AAA guidance software (Siemens Healthcare). The fully automatic registration was found to be satisfactory without further adjustments in 39% of the cases. In the rest of the cases, the registration error was larger than one renal artery diameter or completely manual registration was required.

A recent study from Schwein et al. (2018) [10] including 26 patients who underwent FEVAR assessed the accuracy of fusion technique in guiding visceral vessel cannulation. The results were promising and 83% of the target vessels were cannulated based only on image fusion guidance. However, even in this study the image registration (syngo InSpace 3D/3D fusion; Siemens) was performed in two steps; first automatic registration with focus on the alignment of the bone anatomy, and then a semiautomatic registration emphasizing on the alignment of the calcifications.

The experimental results of our proposed feature-based approach for 3D registration showed acceptable registration error in the majority of cases. The algorithm was evaluated using 14 clinical cases. A limitation of the study was that not all of the preoperative CTs were performed with the same equipment, and the reconstructed MPR images available had a slice thickness that varied from 0.7 mm to 3 mm. However, this represents a real-world clinical situation where preoperative examinations are not always of optimal quality. Even so, the performance of the feature-based algorithm was consistently fair with a maximum average registration error of 3.69 mm and no significant difference in the alignment error of aortic calcifications and bony structures.

## Conclusions

A new and efficient algorithm for 3D3D registration of CTA with CBCT is proposed. The novelty of this work lies in the fact that the algorithm is feature-based whereas commercially available algorithms are intensity-based. Furthermore, the algorithm was validated in a group of clinical cases where image fusion is highly beneficial and is being increasingly used. Our encouraging results require further studies to confirm the clinical usefulness of this algorithm.

## Abbreviations

AAA: Abdominal Aortic Aneurysm
CBCT: Cone Beam Computed Tomography
CTA: Computed Tomography Angiography
EVAR: Endovascular Aortic Repair
