# Correction to: Performance of a feature-based algorithm for 3D-3D registration of CT angiography to cone-beam CT for endovascular repair of complex abdominal aortic aneurysms

**DOI:** 10.1186/s12880-019-0335-3

**Published:** 2019-05-02

**Authors:** Giasemi Koutouzi, Behrooz Nasihatkon, Monika Danielak-Nowak, Henrik Leonhardt, Mårten Falkenberg, Fredrik Kahl

**Affiliations:** 1 Department of Radiology, Institute of Clinical Sciences, Sahlgrenska Academy, Gothenburg, Sweden; 2 K. N. Toosi University of Technology, Tehran, Iran; 3 Department of Electrical Engineering, Chalmers University of Technology, Gothenburg, Sweden; 4 Center for Mathematical Sciences, Lund University, Lund, Sweden

**Correction to: BMC Med Imaging**

**https://doi.org/10.1186/s12880-018-0285-1**

In the original version of this article [1], published on 8 November 2018, there was an error in the name of the 2nd author.

In this correction article the incorrect and correct author name are indicated.

Originally the author name has been published as:

- Behrooz Nasihatkton

The corrected name is:

- Behrooz Nasihatkon
